# Improving ethanol productivity through self-cycling fermentation of yeast: a proof of concept

**DOI:** 10.1186/s13068-017-0879-9

**Published:** 2017-08-02

**Authors:** Jie Wang, Michael Chae, Dominic Sauvageau, David C. Bressler

**Affiliations:** 1 Department of Agricultural, Food and Nutritional Science, University of Alberta, Edmonton, T6G 2P5 Canada; 2 Department of Chemical and Materials Engineering, University of Alberta, Edmonton, T6G 1H9 Canada

**Keywords:** Cellulosic ethanol, Batch, Self-cycling fermentation, Manual cycling fermentation, Ethanol volumetric productivity, Specific productivity, Overall productivity, Annual ethanol productivity, Production cost, Capital cost

## Abstract

**Background:**

The cellulosic ethanol industry has developed efficient strategies for converting sugars obtained from various cellulosic feedstocks to bioethanol. However, any further major improvements in ethanol productivity will require development of novel and innovative fermentation strategies that enhance incumbent technologies in a cost-effective manner. The present study investigates the feasibility of applying self-cycling fermentation (SCF) to cellulosic ethanol production to elevate productivity. SCF is a semi-continuous cycling process that employs the following strategy: once the onset of stationary phase is detected, half of the broth volume is automatically harvested and replaced with fresh medium to initiate the next cycle. SCF has been shown to increase product yield and/or productivity in many types of microbial cultivation. To test whether this cycling process could increase productivity during ethanol fermentations, we mimicked the process by manually cycling the fermentation for five cycles in shake flasks, and then compared the results to batch operation.

**Results:**

Mimicking SCF for five cycles resulted in regular patterns with regards to glucose consumption, ethanol titer, pH, and biomass production. Compared to batch fermentation, our cycling strategy displayed improved ethanol volumetric productivity (the titer of ethanol produced in a given cycle per corresponding cycle time) and specific productivity (the amount of ethanol produced per cellular biomass) by 43.1 ± 11.6 and 42.7 ± 9.8%, respectively. Five successive cycles contributed to an improvement of overall productivity (the aggregate amount of ethanol produced at the end of a given cycle per total processing time) and the estimated annual ethanol productivity (the amount of ethanol produced per year) by 64.4 ± 3.3 and 33.1 ± 7.2%, respectively.

**Conclusions:**

This study provides proof of concept that applying SCF to ethanol production could significantly increase productivities, which will help strengthen the cellulosic ethanol industry.

## Background

The global interest in cellulosic ethanol has surged due to the abundance of feedstock [1], increasing concerns for environmental sustainability and security of energy supplies [2], and the reduction of greenhouse gas emissions compared to first-generation ethanol [3]. Production of cellulosic ethanol requires a pretreatment to open the complex structure of lignocellulosic materials, enzymatic hydrolysis to digest polymers into monomer sugars, microbial propagation to generate inoculum, fermentation of monomer sugars to produce ethanol, and distillation to acquire ethanol. However, according to Chen et al. [4], the cellulosic ethanol industry, as compared to mature first-generation ethanol, is still faced with economic challenges such as high production costs. Therefore, technologies for the production of cellulosic ethanol still need extensive development. Various approaches have been attempted to offset costs, which have been primarily focused on development of effective pretreatment methods to facilitate hydrolysis and fermentation (i.e., efficient sugar digestion and inhibitors reduction, respectively) [5], reduction of enzyme costs/usage [6], and modification/improvement of strains that are efficient in co-fermentation of pentose and hexose sugars under inhibition conditions [7]. Researchers are also working on processing configurations, which are mainly focused on the relationship between hydrolysis and batch fermentation, such as separate hydrolysis and fermentation (SHF), simultaneous saccharification and fermentation (SSF), hybrid hydrolysis and fermentation (HHF), and consolidated bioprocessing (CBP); with SHF and HHF currently being more applicable [8]. Yet, much less effort has been spent on the development of bioprocessing strategies that increase productivity through fermentation methodologies.

Batch operation is a widely used and preferred method for ethanol fermentation [9, 10]. However, batch fermentation incorporates lag and stationary phases, during which ethanol is not being produced at substantial levels. Furthermore, significant downtime is necessary after each fermentation to clean up the reactor and prepare for the next campaign. Thus, one approach to improving productivity of batch fermentation would be to reduce fermentation time and downtime. In addition, to achieve the desired levels of ethanol production, industrial ethanol facilities require a number of large batch bioreactors that operate intermittently to ensure a continuous supply of fermentation product for distillation [11]. Correspondingly, microbial propagation, a lengthy and multi-stage scale-up process that provides fermenters with seed culture, is needed for every batch fermentation cycle [10]. Therefore, batch fermentation and its associated seed cultivation contribute to high capital and operating costs. Altogether, capital and operating costs account for 34 and 33% of the total production costs of cellulosic biofuel, respectively [12]. One approach to address these cost issues is to develop a novel fermentation strategy that will improve productivity.

Self-cycling fermentation (SCF) was developed in the 1990s to facilitate the synchronization of cells. SCF is a semi-continuous cycling process where an online monitoring parameter is used to identify the onset of stationary phase. This identification automatically triggers the removal of half of the fermenter contents, which is immediately replaced with fresh, sterile medium to start a subsequent cycle of growth [13]. Through the operation of SCF, cells are synchronized, which means that all, or almost all the cells are divided at the same time. The actual growth rate of cells will vary depending on growth conditions, which will impact the time required to reach stationary phase, linked to the depletion of a limiting nutrient. Nevertheless, regardless of the time it takes for cells to enter stationary phase, an indicative real-time parameter can be used to trigger the removal and replacement of fermentation broth. Therefore, compared to batch operation, SCF (starting from cycle 2) avoids lag and stationary phases, which means that cells are always in exponential growth, and cycle time equals to generation time [13]. Dissolved oxygen, redox potential, and carbon dioxide evolution rate are commonly monitored parameters in batch reactors, and have all been used as real-time parameters to indicate cell growth and trigger the automation process of SCF [14–16]. Theoretically, SCF can continue indefinitely, with a successful demonstration by Wentworth et al. of more than 100 consecutive cycles for the production of citric acid [14]. Compared to batch fermentation, SCF has also demonstrated increased product yield and/or productivity for many microbial production systems, such as citric acid [14], bioemulsifier [17], shikimic acid [18], and recombinant protein β-galactosidase [19]. Despite these achievements, SCF has not yet been successfully employed for ethanol production. Therefore, considering the relatively low productivity and high production costs associated with batch fermentation of ethanol, we aim to apply SCF to automate the fermentation process and improve productivity for cellulosic ethanol production, thus offsetting high production costs and helping strengthen the cellulosic ethanol industry. The present work provides proof of concept that applying SCF to ethanol fermentation can improve productivity.

## Methods

### Yeast, medium, and inoculum

Superstart™ active distillers dry yeast, *Saccharomyces cerevisiae*, was purchased from Lallemand Ethanol Technology (Milwaukee, WI, USA). The yeast powder was hydrated, and after dilution, cell suspensions were spread on yeast extract peptone dextrose (YPD) agar plates [10 g/L yeast extract (Sigma-Aldrich, St. Louis, MO, USA); 20 g/L peptone (Sigma-Aldrich, St. Louis, MO, USA); 20 g/L d-glucose (Sigma-Aldrich, St. Louis, MO, USA); 14 g/L agar (Thermo Fisher Scientific, Waltham, MA, USA)] and cultivated for 2 days at 30 °C. Individual colonies were transferred to YPD liquid medium (no agar) in glass tubes for overnight cultivation at 30.0 °C and 230 rpm. Some of the overnight culture was transferred to fresh YPD medium to obtain an optical density at 600 nm (OD_600_) of roughly 0.3, and was then allowed to grow under the same conditions until the OD_600_ reached 1.0. The broth was then mixed with 50% (v/v) glycerol at a ratio of 1:1, and stored in vials at −80 °C to produce glycerol stock strains. When required, the stock strain was streaked on a YPD agar plate and allowed to cultivate for 2 days at 30.0 °C, and then stored in a 4 °C fridge. Colonies were transferred to a fresh YPD agar plate monthly.

For all seed cultures and fermentations performed in this work, chemically defined medium was used: 50 g/L d-glucose (Sigma-Aldrich, Oakville, ON, Canada), 6.7 g/L yeast nitrogen base with amino acids (YNB, Sigma-Aldrich, St. Louis, MO, USA), and 0.1 M sodium phosphate buffer (NaH_2_PO_4_·2H_2_O/Na_2_HPO_4_·2H_2_O, Thermo Fisher Scientific, Waltham, MA, USA) at pH 6.0. The medium was filter sterilized (Sartolab™ P20 Plus Filter Systems: 0.2 µm, Thermo Fisher Scientific, Waltham, MA, USA) prior to being used.

To prepare the inoculum, isolated colonies on YPD plates were transferred to 10 mL of chemically defined medium in glass tubes and incubated overnight at 30.0 °C with shaking at 200–250 rpm. A portion of this starter culture was transferred to a 1-L shake flask containing 180 or 600 mL fresh medium to obtain an OD_600_ of ≈0.2 for further incubation under the same condition. When an OD_600_ of ~0.5 was achieved in the shake flask, the culture was used to inoculate the fermentation experiments described below. The inoculum volume for all experiments in the report was ~8% (v/v) of the fermentation medium.

### Dynamic study of yeast fermentation

To baseline the dynamic changes that occur during batch ethanol fermentation using our fermentation system, a total of 24 shake flasks (500 mL) with 270 mL of chemically defined medium (described above) were inoculated with yeast (8%, v/v). The shake flasks were incubated at 30.0 °C with shaking at 200 rpm. Each flask was attached to an S-lock filled with distilled water to minimize air from flowing in the flask and to release gas out of the flask. At eight specific time points, three flasks were taken out of the incubator and sacrificed for analysis, allowing for the analyses to be carried out in triplicate.

### Cycling fermentation

This experiment, in which cycling was performed manually, was designed to mimic the process of SCF, and test whether our fermentation system could result in a stable process of reproducible cycles. The initial cycle had a working volume of 280 mL in a 500-mL shake flask. Additional shake flasks were incubated in parallel to allow for analysis of glucose levels at a given time point. Within fermentation cycles, the additional flasks were taken out from the incubator and monitored to determine the time at which glucose was virtually depleted (<1 g/L; analytical method described below). At this point, experimental flasks were taken out of the incubator and half of the broth volume (140 mL) was manually removed, and immediately replaced with an equal volume of sterile medium to start the next cycle. Immediately after the sterile medium was added, the flask was gently mixed and a 10-mL sample was removed for analysis. This process was repeated until the end of 5th cycle. It should be noted that for each successive cycle, a smaller amount of broth was removed/replaced due to the drop in fermentation broth volume resulting from withdrawal of 10 mL of samples taken for analysis. For example, at the end of cycle 2, there was a total working volume of 270 mL, and thus, only 135 mL were removed/replaced. All shake flasks were capped with an S-lock filled with distilled water. This experiment was performed in triplicate.

### Analytical methods

Optical density (OD_600_) was measured using a spectrophotometer (Ultrospec 4300 pro, Biochrom, England, UK). High OD_600_ values of fermentation broth were diluted with medium to fall within the range of 0.2–0.9, and cell concentration was calculated according to the appropriate dilution factor. The broth pH was measured using a pH meter (Accumet^®^ AB 15, Fisher Scientific, Thermo Fisher Scientific, Waltham, MA, USA). The concentrations of glucose and ethanol were quantified according to Parashar et al. [20]. Briefly, ethanol content was determined by gas chromatography with a flame ionization detector, using 1-butanol as the internal standard. Glucose content was quantified through high-performance liquid chromatography using an HPX-87H column coupled with a refractive index detector. For samples with glucose content lower than 1 g/L or for quick confirmation of glucose depletion during manual cycling fermentation experiments, a Megazyme d-Glucose (glucose oxidase/peroxidase; GOPOD) assay kit (Bray, Ireland) was used, and the whole procedure took no more than 20 min. In this method, glucose concentration was determined through an absorbance reading at 510 nm, which quantified the amount of a quinoneimine dye derived through enzymatic processing of glucose. Samples were filtered (0.22 µm), mixed with GOPOD reagent, incubated at 50.0 °C, and the absorbance reading was compared against both blank and standard samples. Fermentation efficiency was calculated by using the following equation:

$${\text{Fermentation efficiency}}\,{ = }\,\left( {\frac{\text{Amount of ethanol produced}}{\text{Amount of glucose consumed}} \div 0.511} \right) \times 100.$$

Theoretically, 0.511 g of ethanol is produced per gram of glucose. Fermentation samples were examined under a microscope to confirm the lack of bacterial contamination.

### Statistical analysis

Analysis of variance (ANOVA) was conducted with Tukey test set at 95% confidence level by GraphPad Prism 5.04 software (La Jolla, CA, USA). Suspected outliers were evaluated by *Q* test (95% confidence) within triplicate results. The OD_600_ measurement for one of the three flasks examined at the end of cycle 4 of the manual cycling experiment was confirmed as an outlier, and was therefore excluded from our data analyses; the other parameters assessed passed the test and were kept.

## Results

### Dynamic study of batch fermentation

**Fig. 1:**
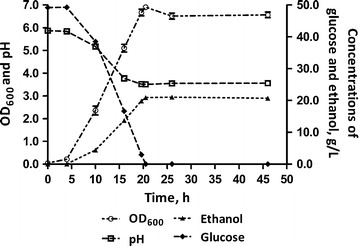
Dynamic study of batch fermentation. Optical density (OD_600_), pH, glucose consumption, and ethanol production were monitored over a 46-h period. *Error bars* represent standard deviation of triplicate experiments

The primary goal of the present work was to explore whether a self-cycling fermentation strategy can be incorporated into ethanol production to improve productivity and/or increase product yield. As a baseline comparison for our system, we first performed batch fermentation, monitoring several parameters (OD_600_, pH, glucose, and ethanol concentrations) at various time points (Fig. 1). During fermentation, glucose, the main carbon source, was consumed by yeast for growth and ethanol production. Glucose was depleted at ~20.5 h, when cell concentration (measured by OD_600_) and ethanol yield reached maximum values. The pH of the fermentation broth dropped while the yeast was growing and stabilized before the onset of stationary phase. The fermentation efficiency at 20.5 h was 86.1 ± 0.4%. Based on the growth curve generated through OD_600_ readings, we estimated the generation time to be approximately 6 h. Inspection of cells under a microscope confirmed that there was no bacterial contamination.

### Cycling study

**Fig. 2:**
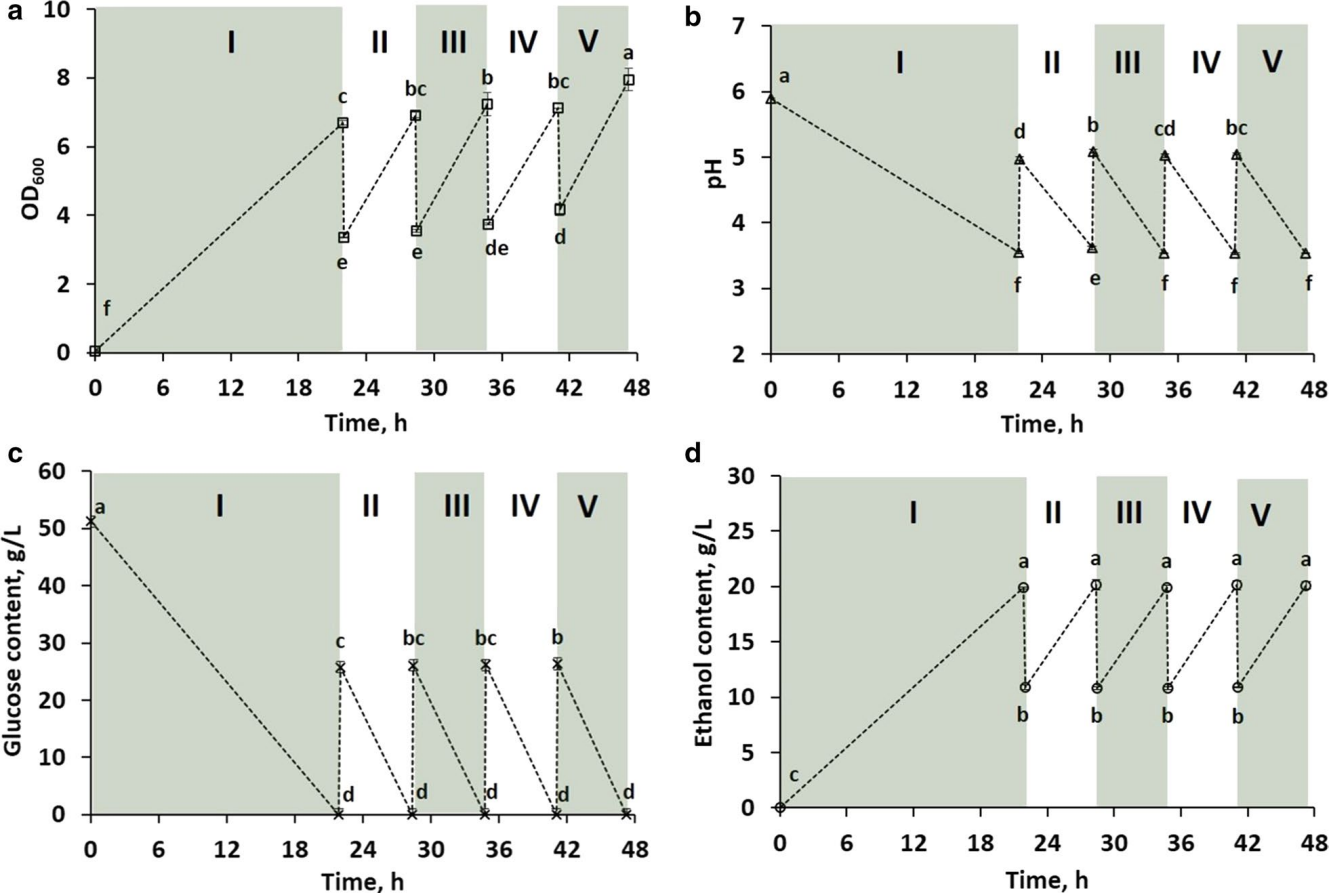
Cycling fermentation experiments. OD_600_ (**a**), pH (**b**), glucose concentration (**c**), and ethanol content (**d**) were monitored through five cycles over a 47-h period. The cycle numbers are indicated with *roman numerals*. *Error bars* represent standard deviation of triplicate experiments, except for the OD_600_ value at 40.9 h in **a** (the end of cycle 4), which shows the result of duplicate samples (see “Methods”). Means that do not share the same letter are statistically different (95% confidence level, Tukey)

Following the batch fermentation experiments, we performed cycling fermentations to determine the impact of incorporating this methodology into an ethanol production system. In this work, cycle time is defined as the time used only for fermentation, excluding the harvest and replacement steps. Once the depletion of glucose had been confirmed, half the fermentation broth was removed and replaced with an equal amount of fresh growth medium, initiating the next fermentation cycle. This was repeated for a total of 5 fermentation cycles. Based on this strategy, in cycle 1, the input content of glucose, as well as the produced amount of ethanol, was roughly twice as much as corresponding values from cycles 2 to 5 (Fig. 2; Table 1). As shown in Fig. 2c, glucose was completely consumed at the end of all cycles. For cycles 2–5, although a smaller amount of ethanol was generated in each cycle compared to cycle 1 (Table 1), the final concentration of ethanol (g/L) was statistically equal in all cycles (Fig. 2d). Additionally, when compared on a per glucose input basis, the yield of ethanol produced was statistically similar in all cycles (Table 1). Thus, the key significance of these experiments is the dramatic decrease in fermentation time required to produce ethanol when the SCF approach was employed. For example, cycle 1 produced 5.6 ± 0.0 g of ethanol in 21.9 ± 0.1 h, while cycles 2, 3, and 4 together produced 7.2 ± 0.2 g of ethanol in 18.8 ± 0.0 h. Thus, cycles 2, 3, and 4 produced 129.2 ± 2.3% of the ethanol generated in cycle 1, but in only 86.0 ± 0.5% of the time.

**Table 1:** Cycling fermentations and overall productivity improvement

Cycle number	Cycle time (h)	Glucose available at onset of cycle (g)	Amount of ethanol produced in a given cycle (g)	Yield of ethanol produced in a given cycle per glucose fed (g/g)	Overall productivity improvement compared to batch (%)
1	21.9 ± 0.1^a^	14.3 ± 0.0^a^	5.6 ± 0.0^a^	0.4 ± 0.0^a^	−9.7 ± 0.6^a^
2	6.4 ± 0.0^b^	6.9 ± 0.1^b^	2.5 ± 0.2^b^	0.4 ± 0.0^a^	15.6 ± 3.4^b^
3	6.3 ± 0.0^bc^	6.8 ± 0.0^c^	2.4 ± 0.1^bc^	0.3 ± 0.0^a^	34.9 ± 2.0^c^
4	6.2 ± 0.0^cd^	6.5 ± 0.0^d^	2.3 ± 0.1^bc^	0.4 ± 0.0^a^	51.1 ± 2.7^d^
5	6.1 ± 0.0^d^	6.3 ± 0.1^e^	2.2 ± 0.1^c^	0.3 ± 0.0^a^	64.4 ± 3.3^e^

Numbers indicate the mean ± standard deviation of triplicate experiments. Within the same column, values with different superscript letters are statistically different

Figure 2b shows that the first cycle started with a pH of 5.9 ± 0.0 and dropped to 3.5 ± 0.1 by the end of the cycle. After the first manual removal and broth replacement, the buffer capacity of the added medium was not strong enough to bring the pH back to the original value, stabilizing at around pH 5.0. For successive cycles, the pH fluctuated roughly from pH 5.0 at the beginning of the cycles to pH 3.5 at their ends. In terms of yeast growth (Fig. 2a), cultures from all cycles ended with a statistically similar optical density, except for cycle 5, which generated a statistically higher value than the other cycles. However, the starting OD_600_ values of cycles generally increased as a function of cycle number, with cycle 5 being the highest among all cycles. Furthermore, when the change in OD_600_ was compared among cycles 2–5, there was no significant difference.

**Fig. 3:**
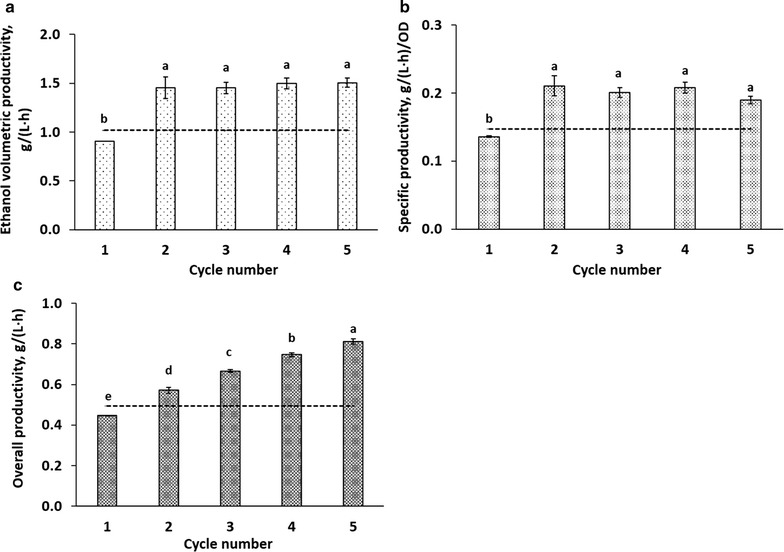
Productivities of cycling fermentation experiments. Ethanol volumetric productivity (**a**), specific productivity (**b**), and overall productivity (**c**) were determined for manual cycling experiments. The *horizontal solid line* represents the mean values obtained through a dynamic batch study (Fig. 1), where ethanol production reached a plateau at ~20.5 h. *Error bars* represent standard deviation of triplicate experiments, except for cycle 4 in **b**, which represents duplicate samples (see “Methods”). Means that do not share the same letter are statistically different (95% confidence level, Tukey)

Ethanol volumetric productivity (Fig. 3a) represents the ethanol produced (g/L) at each cycle per corresponding cycle time. Compared to the 1st cycle, which is essentially a normal batch fermentation, successive manual cycling significantly improved ethanol volumetric productivity (Fig. 3a). For example, cycle 2 displayed an ethanol volumetric productivity increase of 60.4 ± 12.1 and 43.1 ± 11.6%, compared to cycle 1 and batch fermentation, respectively (Fig. 1). Specific productivity (Fig. 3b)—representing the ethanol volumetric productivity per biomass content (based on OD_600_ readings)—was 55.1 ± 9.7 and 42.7 ± 9.8% greater in cycle 2 than in the first cycle and batch fermentation, respectively. These values did not differ significantly in cycles 2–5. To obtain an approximation of the influence of self-cycling fermentation on overall production efficiency, we calculated the overall productivity based on the laboratory conditions used (Fig. 3c). Overall productivity for a cycle considers the ethanol (g/L) accumulated at the end of the cycle per total process time—which includes medium preparation, the cumulative fermentation cycle time, as well as the time required for the harvesting and refilling steps (3 min each in lab conditions). For a single-batch fermentation, medium preparation, sterilization of media and equipment, and seed cultivation took a total of 21.8 h, while slightly longer was spent for manual cycling fermentation runs (22.7 h); this increase was due to longer time necessary for filter sterilization of a larger volume of medium and a longer period of seed culture cultivation. The length of batch fermentation (20.5 h) was adapted from our dynamic study as similar laboratory procedures, reagents, and glassware were used for both. For the total process, 42.2 ± 0.0 and 69.9 ± 0.1 h were spent for batch and manual cycling fermentation (5 cycles), respectively, which means that manual cycling for 5 fermentation cycles took 65.4 ± 0.2% longer than batch. Compared to batch, an increase of cycle number in manual cycling fermentation significantly improved overall productivity (Fig. 3c), and a 64.4 ± 3.3% improvement was observed when 5 cycles were involved (Table 1).

### Annual ethanol productivity for potential scale-up

To appreciate how implementation of a self-cycling strategy could potentially impact annual ethanol production goals at large scale, we compared SCF with batch in terms of annual ethanol productivity, which represents the ethanol produced per year (*P*, ton/year). Feng et al. determined the annual fermentation operation time (*t*_annual_) for an ethanol plant to be 7920 h (330 days) [21]. For a reactor with a working volume (*V*) of 10^5^ L, downtime between cycles was estimated at 6.0 h (*t*_d-batch_) and 0.25 h for batch and SCF methodology, respectively [21]. Residence time (*t*_f-batch_, *t*_f-SCF_) and ethanol produced (*C*_batch_, *C*_SCF_) per campaign for batch and SCF were adapted from our dynamic batch and manual cycling studies (cycles 1 and 2), respectively. For demonstration of the calculations below, mean values of triplicate experimental results were used.

For batch fermentation, the number of campaigns (*N*_batch_) possible in a year would be:

$$\begin{aligned} N_{{\text{batch}}} = & \;\frac{{t_{{\text{annual}}} }}{{t_{\text{f-batch}} + t_{\text{d-batch}} }} \\ = &\; \frac{{7920\;{\text{h/year}}}}{{20.5\;{\text{h/campaign}}\;{ + }\; 6. 0\;{\text{h/campaign}}}} \\ = &\; 299\;{\text{campaign/year}} .\\ \end{aligned}$$

Thus, the annual ethanol productivity for batch fermentation (*P*_batch_) would be:

$$\begin{aligned} P_{\text{batch}} = &\; N_{\text{batch}} \times C_{\text{batch}} \times \,V \\ = & \; 299\;{\text{campaign/year}}\, \times \, 2 0. 9\,{\text{g/L/campaign}}\, \times \,10^{5} \,{\text{L}} \\ { = } & \; 6. 2 5\, \times \;10^{8} \;{\text{g/year}}\; \\ = & \;625\,{\text{ton/year}} .\\ \end{aligned}$$

We assume that a plant can continuously run SCF for *x* (*x* ≥ 1) cycle numbers (with 0.25 h downtime between cycles) each campaign, after which point, the reactor will need to be cleaned and set up for a new campaign (6 h downtime following the last SCF cycle; assumed to be the same as batch).

For a single SCF campaign, the total of residence time (*t*_f-SCF_) would be the sum of all *x* cycles:

$$\begin{aligned} t_{\text{f-SCF}} = & \; t_{\text{f-cycle 1}}\,+\, \left( {t_{\text{f-subsequent cycles}} } \right)\left( {x - 1} \right){\text{ cycles}}\; \\ { = } & \; 2 1. 9\,{\text{h}}\, + \,\left( {6.4\,{\text{h/cycle}}} \right)\left( {x - 1} \right){\text{ cycles}} \\ { = } & \; 1 5. 5\,{\text{h}}\,+\, 6.4x\,{\text{h}} .\\ \end{aligned}$$

Similarly, the total downtime (*t*_d-SCF_) for a single SCF campaign can be summarized as follows:

$$\begin{aligned} t_{\text{d-SCF}} = &\; \left( {t_{{{\text{d-}}\left( {x - 1} \right){\text{ cycles}}}} } \right)\left( {x - 1} \right){\kern 1pt} \;{\text{cycles}}\;{ + }\;t_{{\text{d-cycle}\,{x}}} \\ = &\; \left( {0.25\,{\text{h/cycle}}} \right)\left( {x - 1} \right)\;{\text{cycles}}\,{ + }\,6.0\,{\text{h}} \\ =&\; \, 5. 7 5\,{\text{h}}\,{ + }\,0.25x\,{\text{h}} .\\ \end{aligned}$$

Therefore, the total number of SCF campaigns that can be run each year (*N*_SCF_) can then be determined as follows:

$$\begin{aligned} N_{\text{SCF}} = &\, \frac{{t_{\text{annual}} }}{{t_{\text{f-SCF}} + t_{\text{d-SCF}} }} \\ = &\, \frac{{7920\,{\text{h/year}}}}{{{{\left[ {\left( {15.5\,{\text{h}}\,+\, 6 . 4x\,{\text{h}}\,} \right) + \left( {5.75\;{\text{h}}\,+\,0.25x\,{\text{h}}} \right)} \right]} \mathord{\left/ {\vphantom {{\left[ {\left( {15.5\,{\text{h}}\,{ + 6} . 4x\,{\text{h}}\;} \right) + \left( {5.75\;{\text{h}}\;{ + }\;0.25x\;{\text{h}}} \right)} \right]} {\text{campaign}}}} \right. \kern-0pt} {\text{campaign}}}}} \\ = &\, \frac{{7920\,{\text{h/year}}}}{{{{\left[ {21.25\,{\text{h}}\, + \,6.65x\;{\text{h}}} \right]} \mathord{\left/ {\vphantom {{\left[ {21.25\,{\text{h}}\,+ \,6.65x\,{\text{h}}} \right]} {\text{campaign}}}} \right. \kern-0pt} {\text{campaign}}}}}. \\ \end{aligned}$$

Using the SCF strategy, the total amount of ethanol produced per campaign with *x* cycles (*E*_SCF_) can be calculated as shown below:

$$\begin{aligned} E_{\text{SCF}} = &\, \left( {C_{\text{f-cycle 1}} } \right)\left( V \right)\, + \,\left( {C_{\text{f-subsequent cycles}} } \right)\left( V \right)\left( {x - 1} \right)\;{\text{cycles}} \\ { = } & \,V \times \;{{\left[ {\left( {C_{\text{f-cycle 1}} } \right) + \left( {C_{\text{f-subsequent cycles}} } \right)\left( {x - 1} \right)\;{\text{cycles}}} \right]} \mathord{\left/ {\vphantom {{\left[ {\left( {C_{\text{{f-cycle 1}}} } \right) + \left( {C_{\text{{f-subsequent cycles}}} } \right)\left( {x - 1} \right)\;{\text{cycles}}} \right]} {\text{campaign}}}} \right. \kern-0pt} {\text{campaign}}} \\ = &\,10^{5} \,{\text{L}} \times {{\left[ {19.9\,{\text{g/L}} + \,\left( {9.3\,{\text{g/L/cycle}}} \right)\left( {x - 1} \right)\;{\text{cycles}}\;} \right]} \mathord{\left/ {\vphantom {{\left[ {19.9\,{\text{g/L}} + \;\left( {9.3\,{\text{g/L/cycle}}} \right)\left( {x - 1} \right)\;{\text{cycles}}\;} \right]} {\text{campaign}}}} \right. \kern-0pt} {\text{campaign}}} \\ = &\, 10^{5} \,{\text{L}} \times \;{{\left[ {10.6\,{\text{g/L}} + \;9.3x\,{\text{g/L}}} \right]} \mathord{\left/ {\vphantom {{\left[ {10.6\,{\text{g/L}} + \;9.3x\,{\text{g/L}}} \right]} {\text{campaign}}}} \right. \kern-0pt} {\text{campaign}}}. \\ \end{aligned}$$

Therefore, the annual ethanol productivity (*P*_SCF_) would be:

$$\begin{aligned} P_{\text{SCF}} = N_{\text{SCF}} \times E_{\text{SCF}} = &\, \frac{{7920\;{\text{h/year}}}}{{{{\left[ {21.25\;{\text{h}}\;{ + }\; 6. 6 5x\;{\text{h}}} \right]} \mathord{\left/ {\vphantom {{\left[ {21.25\;{\text{h}}\;{ + }\; 6. 6 5x\;{\text{h}}} \right]} {\text{campaign}}}} \right. \kern-0pt} {\text{campaign}}}}} \times 10^{5} \;{\text{L}} \\ &\times \;{{\left[ {10.6\;{\text{g/L}}\;{ + }\;9.3x\;{\text{g/L}}} \right]} \mathord{\left/ {\vphantom {{\left[ {10.6\;{\text{g/L}}\;{ + }\;9.3x\;{\text{g/L}}} \right]} {\text{campaign}}}} \right. \kern-0pt} {\text{campaign}}} \\ = &\, \frac{{792 \times \left( {10.6\; + \;9.3x} \right)}}{21.25\; + \;6.65x}\,{\text{ton/year}} .\\ \end{aligned}$$

**Fig. 4:**
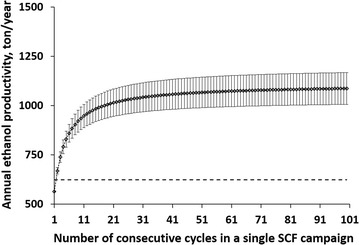
Annual ethanol productivity. Annual ethanol productivity was calculated assuming the number of consecutive cycles operated for each SCF campaign could range from 1 to 100. The *horizontal solid line* represents the mean values obtained through a dynamic batch study (Fig. 1), where ethanol production reached a plateau at ~20.5 h. *Error bars* were calculated from the errors in ethanol yield and cycle time of SCF cycles and represent standard deviation of triplicate experiments

If cycling fermentation is operated for 5 consecutive cycles (*x* = 5), as was the case in our manual cycling study, *P*_SCF_ would be 830 ± 41 ton/year, representing a 33.1 ± 7.2% improvement in annual ethanol productivity compared to batch (*P*_batch_, 624 ± 3 ton/year). As implied by Fig. 4, as the number of consecutive cycles (*x*) in each SCF campaign increases, the annual ethanol productivity (*P*_SCF_) initially increases sharply before the increase becomes almost negligible (as the fraction of downtime to production time becomes negligible). Moreover, annual ethanol productivity in SCF (*P*_SCF_) is expected to be significantly greater than that of batch fermentation (*P*_batch_), even when only 2 cycles (*x* ≥ 2) are operated for each SCF campaign.

Examined from a different perspective, the goal of SCF application may be to achieve the same annual ethanol productivity as batch fermentation (based on 625 ton/year), but in a shorter amount of time (i.e., fewer campaigns). Using the equation above, if SCF operation is based on 5 consecutive cycles (*x* = 5), each SCF campaign will produce 5.7 tons of ethanol (*E*_SCF_). Therefore, the number of SCF campaigns required to produce 625 tons of ethanol through SCF is roughly 110 per year (*P*_batch_/*E*_SCF_). Given that each SCF campaign would require a total of 54.5 h (*t*_f-SCF_ + *t*_d-SCF_), the total time required for 110 campaigns is roughly 6000 h. This is approximately 1900 h (~80 days) shorter than the annual fermentation time required for batch fermentation to produce the same amount of ethanol.

## Discussion

### Dynamic study

Ethanol production is tightly associated with cell growth. As such, when the limiting nutrient is depleted under anaerobic conditions, yeast stops growing and producing ethanol, entering into stationary phase. In the present study, the ethanol titer was lower than what has been achieved in industry [10]; this is because a defined medium (6.7 g/L YNB, 0.1 mol/L phosphate buffer, and 50 g/L glucose), where glucose was the main carbon source, was used at a low concentration, instead of directly using a typical hydrolysate of lignocellulosic material that contains a mixture of pentose and hexose sugars, corn steep liquor, and inhibitors [10]. This was done in order to simplify the implementation of SCF operation for the study at hand.

As is typical during ethanol fermentation, the pH of our batch system decreased, likely because the uptake of buffering materials such as amino nitrogen compounds, the excretion of organic acids [22], the utilization of ammonium—which releases hydrogen ions outside of the cell [23]—and the production of carbonic acid due to the reaction of carbon dioxide (released by yeast) with water. Nevertheless, given the data on glucose consumption and ethanol production in batch fermentation (Fig. 1), the medium system was adequately buffered and was able to achieve relatively high fermentation efficiency and biomass yield. It should be noted that the fermentation efficiency observed in the batch fermentation is lower than those typically observed in fermentations using wheat grain as feedstock (roughly 90–94%) [20, 24]. One explanation for this may be the presence of oxygen in the headspace of shake flasks, which would enable yeast to momentarily grow aerobically to produce biomass, rather than ethanol. Furthermore, the medium used was not optimized for ethanol production, as is the case with ethanol fermentations using grains. Despite the suboptimal conditions, the fermentation efficiency still reached 86.1 ± 0.4%. Furthermore, we are exploring the use of other media that could be employed in SCF operation to further improve fermentation efficiency.

### Cycling study

Since Fig. 1 revealed that the onset of stationary phase was tightly linked to the depletion of glucose, identification of the specific time point where glucose is depleted is important for SCF systems. Doing so may allow for harvest and fresh medium addition right as cells would enter stationary phase, where ethanol production ends and cell metabolism begins to change. As suggested in the manual cycling study, sugar was depleted by the end of each cycle (Fig. 2c), which would help bioethanol producers avoid unnecessary sugar losses and improve process economics. This also gives SCF an advantage over chemostat operation, where some of the nutrients are washed out throughout the process.

It should be noted that, for cycles 2–5, there was a slight gradual increase in starting cell concentration (Fig. 2a), yet no significant difference in OD_600_ change was found among the four cycles. This is possibly due to the settling of cells during manual broth removal, which made the cell concentration of removed sample slightly lower than that of the broth left inside of shake flasks. The settling might be the reason why one of the three samples at the end of cycle 4 was rejected as an outlier (*Q* test) of its parallel samples in cell concentration (as measured by OD_600_). Whereas the measurements of the other parameters (pH, glucose, and ethanol concentrations) used techniques that are not related to cell concentration and the values were retained by *Q* test, we still incorporated that sample for the results of parameters not based on OD_600_. All in all, this settling phenomenon will likely not be an issue in scale-up due to continual stirring during broth removal.

It should also be noted that due to the sampling required for analysis, the fermentation broth volume decreased by 10 mL in each cycle, which led to a reduction in total glucose input (g) and also the total amount of ethanol produced (g) from cycles 1 to 5 (Table 1). While such sampling may have slightly decreased total ethanol production in our shake flask studies, this would not be significant in bioreactor operation, as sampling volumes are negligible in larger vessels. Nevertheless, our data clearly provide proof of concept that our SCF approach to ethanol production can retain ethanol yield and increase ethanol yield per fermentation time.

As shown in the cycling experiments, which mimicked SCF, at the end of each cycle, half of the cell population was harvested with the other half serving as the “inoculum” (50% (v/v) of the working volume) for the next cycle. SCF can contribute several benefits to the ethanol production process.

Firstly, in current cellulosic ethanol plants, a few steps are typically required to gradually scale-up a seed culture for inoculation, which is a common practice for batch fermentation [10]. Whereas for SCF operation, once inoculated for the first cycle, the yeast propagation process is no longer required for subsequent cycles, and is only necessary when a new SCF campaign is initiated. Thus, the more cycles incorporated into an SCF campaign, the fewer microbial propagation steps would be required. This will save nutrients, energy, and work hours spent on the propagation stage. Furthermore, the cycling strategy of SCF is easily compatible with current processing infrastructure, since the removed volume of broth can be fed continuously into a distillation column, and fresh medium could be pumped from the hydrolysis section (SHF) of an integrated process.

Secondly, as shown in the cycling experiments (Table 1), compared to batch operation, fermentation time is dramatically reduced in SCF, without compromising the ethanol yield. This is likely because the lag and stationary phases are removed from SCF operation [13], and therefore, cells are always in exponential growth. It should be noted that only two data points are shown for each cycle (Fig. 2), so there is a possibility that the substrate was depleted earlier than reported, which could make the fermentation cycle times shorter and productivity higher. Also, cycle times varied among cycles 2–5 (Table 1). These cycle times are based on the confirmation of glucose disappearance (using the GOPOD method) from additional shake flasks incubated in parallel to minimize volume change of the main experimental flasks and avoid exposure to air during fermentation. Thus, this analytical procedure may have introduced a slight delay, and the cycling times reported may not be absolutely reflective of what happened in the main experimental flasks. Furthermore, although some of the cycling times were statistically different, they were only different by a few minutes. In the implementation of a fully automated SCF system, the overestimation of cycle time is unlikely, since the fermentation will be monitored by a real-time parameter, which will automatically trigger the cycling process once cells enter stationary phase.

Finally, for cellulosic ethanol production, the pretreatment of feedstocks can form or release inhibitors, such as furfural, phenolic compounds, and weak acids, that can inhibit cell growth and ethanol production [8]. It has been reported that inhibition can be biochemically mitigated through exposing microbe seed cultures to inhibitors during propagation [25]. Therefore, for SCF, it may be worthwhile in the future to test whether the “inoculum” (i.e., half of the fermentation broth from the previous cycle), which has been grown in the presence of any potential inhibitors, will help the following cycle achieve better inhibitor tolerance and therefore better ethanol production.

According to Table 1, the yield of ethanol produced per glucose fed was statistically similar for all cycles. Thus, the 43.1 ± 11.6% improvement in ethanol volumetric productivity (g/(L h)) observed in the cycling fermentation study was due to the reduced fermentation time. This result, even though performed in shake flasks—which may lead to higher variability than controlled bioreactors, is still consistent with those of reported SCF systems—where bioreactors were used and improvements in productivity were achieved primarily due to shorter fermentation time than batch [17, 19]. It should be noted that cell synchrony was not assessed in this study as synchrony has been shown to be established after 5–10 SCF cycles. Therefore, the reduction of fermentation time in the present study is unlikely to be due to cell synchrony. Whichever is the case, significant improvements in productivities are observed and optimization of cell synchronies could possibly further enhance these results. This supports the argument that application of SCF in industrial ethanol production may reduce the fermentation time necessary to reach current production goals, without changing existing infrastructures. The reduction of fermentation time leads to lower operation costs, which currently make up 33% of total production costs [12]. Alternatively, this improvement also suggests that current production levels could be met by employing smaller bioreactors in an SCF strategy. In this way, new cellulosic ethanol plants may be able to reduce their capital costs, which typically account for 34% of the total production cost [12]. Given the similar biomass yields of all cycles (Fig. 2a), the specific productivity of all cycles is clearly most impacted by the ethanol volumetric productivity. Again, this strongly suggests that the improvement in specific productivity is mainly due to the reduced cycle time. Overall productivity in a laboratory setting indicates how the cycling strategy could impact the overall process. Examining the cycling process in a single shake flask, results support that when more cycles are incorporated in a campaign, higher overall productivity is achieved (Fig. 3c). Note that there was a 9.7 ± 0.6% reduction in overall productivity compared to batch when cycle 1 (essentially a batch cycle) was performed. This probably results from the extra time required to confirm the disappearance of glucose in parallel flasks prior to performing the manual cycling, which results in a slight overestimation of cycling times in the manual cycling study. In dynamic batch study, flasks were directly taken out from the incubator and sacrificed for dynamic analysis throughout the fermentation process. In addition, this difference could result from batch to batch variations as batch operation is known to be variable [17].

### Annual ethanol productivity

Currently, cost reductions of cellulosic ethanol production primarily come from improvements in pretreatment [5], hydrolysis [6], and strain improvement [7]. However, much less effort has been spent on improving productivity and reducing costs by directly changing processing strategies of fermentation. To get an idea whether applying cycling strategies to ethanol fermentation could increase the total amount of ethanol that could be produced per year (annual ethanol productivity) at large scale, we assumed that, with the exception of the length of downtime, SCF would operate under the same conditions as batch. According to Feng et al. downtime between cycles is approximately 6 h for batch fermentation [21], which includes the time used to harvest broth, clean, sterilize, and refill the reactor. However, only 0.25 h will be needed to exchange volumes between SCF cycles [21], since only half the volume of the broth will be harvested, and no cleaning or sterilization steps are necessary between cycles. Furthermore, the time required to add fresh medium to the reactor will actually be part of the cycle time because cells continue to grow as soon as the nutrients are added to the reactor. These calculations indicate that, compared to batch operation, if a five-cycle SCF strategy were implemented for each campaign performed at a plant, either the amount of ethanol produced annually would be greatly increased or annual fermentation time would be dramatically reduced for the same production level. These improvements, which would help reduce production cost, are mainly attributable to the reduced fermentation time, as well as the reduced fraction of downtime.

While theoretically SCF can run indefinitely, there is a concern that in long-term continuous operation of SCF, a non-beneficial mutation or severe bacterial contamination may occur and affect ethanol productivity. This can be averted by implementing SCF operation with number of cycles (*x*) that is low enough to minimize the probability of mutations or contamination affecting productivity, but large enough to significantly increase annual ethanol productivity. Figure 4 provides a basis for the determination of an optimal number of cycles. Based on these results, we found that, with regard to annual ethanol productivity, operation of SCF for approximately 20 sequential cycles (essentially 20 generations) would provide a good balance between improved productivity and reduced risks of mutation/contamination.

To the best of our knowledge, the present study is the first to provide proof of concept that SCF could be employed for ethanol production towards elevated productivities. Feng et al. attempted to implement SCF operation by using redox potential as a feedback control parameter for ethanol fermentation [21]. Air was purged in the reactor when the redox potential of the broth fell below a certain level, so that redox potential could generate a transient response. However, this switch between anaerobic and aerobic conditions during fermentation likely disrupted cell metabolism, and thus affected ethanol production. Therefore, this artificial manipulation of redox potential during SCF for ethanol production led to longer fermentation time and reduced ethanol volumetric productivity compared to batch operation.

## Conclusions

By mimicking the SCF process in manual cycling experiments at the shake flask scale, the required fermentation time was greatly reduced, while maintaining statistically equivalent glucose to ethanol conversion. With respect to batch operation, our cycling strategy improved ethanol volumetric productivity by 43.1 ± 11.6%, overall productivity by 64.4 ± 3.3%, and estimated annual ethanol productivity by 33.1 ± 7.2%. These elevated productivities may lead to reduced capital costs (i.e., the number and/or size of fermenters required) or operation costs (i.e., the fermentation time required), increased amounts of ethanol production per year, and could eventually lower production costs, relative to batch fermentation. This work, even though performed under suboptimal conditions, has successfully provided proof of concept that adoption of an SCF strategy for cellulosic ethanol could increase productivities, thereby opening up a great possibility for applying novel cycling fermentation strategies to strengthen the cellulosic ethanol industry.

## Abbreviations

SCF: self-cycling fermentation
SHF: separate hydrolysis and fermentation
SSF: simultaneous saccharification and fermentation
HHF: hybrid hydrolysis and fermentation
CBP: consolidated bioprocessing
GOPOD: glucose oxidase/peroxidase
OD_600_: optical density at 600 nm
YNB: yeast nitrogen base with amino acids
YPD: yeast extract peptone dextrose
*P*_batch_, *P*_SCF_: annual ethanol productivity (ton/year) for batch and SCF, respectively
*t*_annual_: annual fermentation operation time, h
*t*_f-batch_, *t*_d-batch_: for a single batch campaign, the residence time (h) and downtime (h), respectively
*C*_batch_, *C*_SCF_: ethanol titer produced per cycle (g/L) in batch and SCF, respectively
*N*_batch_, *N*_SCF_: the number of campaigns possible in a year for batch and SCF, respectively
*V*: working volume, L
*x*: for a single SCF campaign, assumed numbers of cycles that a plant can continuously run
*t*_f-cycle 1_, *C*_f-cycle 1_: for the first cycle of an SCF campaign, the residence time (h) and the titer of ethanol produced (g/L), respectively
*t*_f-subsequent cycles_, *C*_f-subsequent cycles_: for cycles following the first one in an SCF campaign, the residence time of the cycle (h) and the titer of ethanol produced (g/L), respectively
*t*_d-(*x* − 1) cycles_: down time (h) for each cycle in a single SCF campaign, except cycle *x*
*t*_d-cycle *x*_: down time for cycle *x* (the last cycle) in a single SCF campaign
*t*_f-SCF_, *t*_d-SCF_, *E*_SCF_: for a single SCF campaign with *x* cycles, the total residence time (h), the total downtime (h), and the total amount of ethanol produced (g), respectively
